# Gender Differences in the Protective Effects of Social Participation on Depressive Symptom Trajectories Among Middle-Aged and Older Adults in China: A Nationwide Longitudinal Study

**DOI:** 10.3390/healthcare14131845

**Published:** 2026-06-25

**Authors:** Weiwei Huang, Yingxuan Wu, Xinyu Yan, Xiaoning Hao

**Affiliations:** 1 School of Public Health, Guangxi Medical University, Nanning 530021, China; 2 Division of Health Security Research, China National Health Development Research Center, National Health Commission, Beijing 100044, China

**Keywords:** social participation, depressive symptoms, gender differences, GBTM, middle-aged and older adults

## Abstract

**Highlights:**

**What are the main findings?**

- Social participation showed a significant relationship with the longitudinal trajectories of depressive symptoms.
- Substantial gender heterogeneity was observed in how social participation buffered against adverse depressive trajectories.

**What are the implications of the main findings?**

- Social participation plays a positive role in alleviating depressive symptoms.
- Gender-sensitive intervention strategies help promote mental well-being among middle-aged and older adults.

**Abstract:**

**Background/Objectives**: Against the background of the rapid aging of the population, the symptoms of depression are a major health problem for middle-aged and older adults. This study analyzes the relationship between social participation and the trajectory of depressive symptoms and whether this association varies by gender. **Methods**: The data comes from five rounds of surveys conducted by the China Health and Retirement Longitudinal Study (CHARLS) from 2011 to 2020, including a total of 5796 participants aged 45 or above. The depressive symptoms of each wave are measured using the 10-item Center for Epidemiologic Studies Depression Scale (CESD-10). Social participation was defined as the number of reported activities (0, 1, or ≥2). The development trajectory of depressive symptoms was analyzed through Group-Based Trajectory Modeling (GBTM), and their links with social participation level were subsequently quantified using multinomial logistic regression. Gender differences were assessed via interaction tests and stratified models. **Results**: GBTM identified four distinct depressive symptom trajectories: low (29.71%), moderate (42.72%), increasing (22.07%), and high (5.50%). Compared with no participation, engaging in one activity was linked to lower odds of falling into the moderate, increasing, and high trajectories; the association was stronger for ≥2 activities. Gender-stratified analyses revealed substantial heterogeneity (all interaction *p* < 0.01). Among women, single-activity participation was associated with lower odds across all three adverse trajectories. Among men, similar associations required ≥2 activities, with single-activity participation linked only to lower odds of the high trajectory. **Conclusions**: Higher levels of social participation have significantly reduced the depressive symptoms of middle-aged and older adults, and the gender differences are pronounced. Interventions should improve access to social participation for older women and promote activity diversity for older men.

## 1. Introduction

Against the backdrop of global population aging, depressive symptoms in middle-aged and older adults have become an urgent public health problem. They are usually characterized by persistent low mood and decreased interest, accompanied by dysfunctions such as impaired attention, reduced sleep quality and decreased appetite. The detrimental consequences of depressive symptoms are particularly pronounced among middle-aged and older adults: such as impaired performance of daily life [1], falls and injurious falls [2], incident cardiovascular disease [3,4], dementia [5] and mild cognitive impairment [6], as well as increased all-cause mortality [7], collectively placing a heavy load on individuals, their families, and the wider health-care system.

The Comprehensive Mental Health Action Plan for 2013–2020 issued by the World Health Organization (WHO) points out that there is a high risk of mental health problems among the elderly. Insufficient identification of depressive symptoms and lack of intervention have always been a persistent problem. The challenge is acute in China, which is undergoing rapid demographic aging. By the end of 2025, China’s population aged 60 and above will reach 323.38 million, accounting for 23.0% of the total population [8]. In parallel, the detection rate of depression symptoms in people aged 45 and above in China is 32.62%, with a markedly higher prevalence in females (39.53%) than in males (25.23%) [9]. Faced with these realities, pinpointing modifiable protective factors that can postpone or forestall the emergence and worsening of depressive symptoms in mid-to-late adulthood has become a key priority on the aging-health research agenda.

Defined as taking part in structured activities embedded in community or other social settings, social participation is broadly regarded as a low-cost, readily accessible, and non-pharmacological pathway for enhancing health among those involved [10]. In the mid-to-late adult population, social participation is characterized by an emphasis on community-based activities and interpersonal interactions [11], including recreational activities (e.g., dancing, playing chess or mahjong), community engagement (e.g., volunteering, community-organized events), and informal social interactions (e.g., socializing with friends or neighbors), with overall participation levels tending to decline with advancing age [12]. As this population gradually withdraws from formal professional roles after retirement, retirees with high social participation tend to have higher life satisfaction and less negative emotions [13]. The types of social participation of middle-aged and older adults have been shown to have different effects on their physical, mental health and cognitive health [10]. Regular participation in society can effectively slow down their weak development [14].

Social participation is one of the pillars of active aging and has long been regarded as a key guarantee for mental health in later life. In 2002, the Madrid International Plan of Action on Ageing adopted by the United Nations emphasized that sustained engagement in social activities is of particular importance for safeguarding psychological well-being across mid-life and beyond [15]. Activity theory similarly posits that older adults can establish new social roles through social participation, thereby mitigating the depressive symptoms precipitated by role transitions [16]. Many studies have found a close relationship between social participation and depressive symptoms [17], and more social participation reduces the risk of depression in late life [18], and such participation exerts a sustained ameliorative effect on these symptoms [19]. Conversely, depressive symptoms erode individuals’ willingness to engage socially, thereby giving rise to a vicious cycle that progressively exacerbates the depressive state [20]. Nevertheless, whether these protective effects apply uniformly across all population subgroups warrants further scrutiny, with gender differences being especially worthy of attention.

Data from the Organisation for Economic Co-operation and Development (OECD) indicate that older women generally experience lower socioeconomic status and constrained financial resources [21], which structurally limits their access to social participation, whereas older men tend to sustain a relatively stable social identity through more frequent engagement in formal social activities [22]. Traditional gender-based divisions of labor lead to an abrupt contraction of females’ social roles upon retirement, while older females continue to shoulder caregiving responsibilities, thereby bearing multifaceted stress burdens [23]. A study drawing on China-specific data further revealed that the overall level of social participation among older females was lower than that of older males, with this gender gap widening progressively with advancing age [24]. Moreover, the mechanisms through which social participation reduces depression risk exhibit notable gender heterogeneity: emotional social support plays a more prominent mediating role among females [25], whereas males rely more heavily on instrumental social support [26].

While a large amount of the literature has probed how social engagement relates to depression, important blind spots persist. To begin with, most prior work has relied on cross-sectional data and is therefore unable to observe the dynamic evolution of depressive symptoms over time; even among longitudinal studies, most have relied on no more than three or four waves of follow-up data, which constrains the precision and stability of trajectory identification. Secondly, most existing studies have treated social participation as a binary variable (participant vs. non-participant) or classified it into several activity types, thus ignoring the potential dose–response relationship between participation and depressive outcomes. Third, whether gender moderates the longitudinal relationship between social participation and the trajectory of depressive symptoms has not been systematically examined. Overlooking this dimension may result in systematic bias when evaluating the effectiveness of intervention strategies, owing to the neglect of gender heterogeneity, particularly in the Chinese context where gender disparities in social role norms are pronounced. Fourth, much of the available evidence has centered on older adults, with limited attention to people aged 45 and above, but the trajectories of depression and social participation patterns are undergoing significant changes in this population.

In response to these gaps, the present study draws on five waves of follow-up data spanning ten years from the China Health and Retirement Longitudinal Study (CHARLS, 2011–2020). First, Group-Based Trajectory Modeling (GBTM) was applied in an exploratory manner to delineate latent developmental courses of depressive symptoms among middle-aged and older adults aged 45 and above, allowing heterogeneous developmental patterns to emerge naturally from the data rather than relying on a priori groupings. Second, grounded in the theoretical frameworks outlined above, specifically activity theory and gender-differentiated social support mechanisms, social participation was operationalized as an ordinal variable (0, 1, or ≥2 activities) to capture potential gradient effects, and the links between the count of social activities and depressive symptom trajectories, together with their gendered variation, were then tested in a confirmatory mode. These confirmatory objectives and the expected directions of effects were established independently of and prior to the trajectory solution obtained from GBTM. The results of this study can not only enrich the theoretical views on gender differences in mental health in old age, but also identify high-risk groups with depressive symptoms, and provide empirical evidence for the development of gender-sensitive mental health interventions.

## 2. Materials and Methods

### 2.1. Data Source

**Figure 1:**
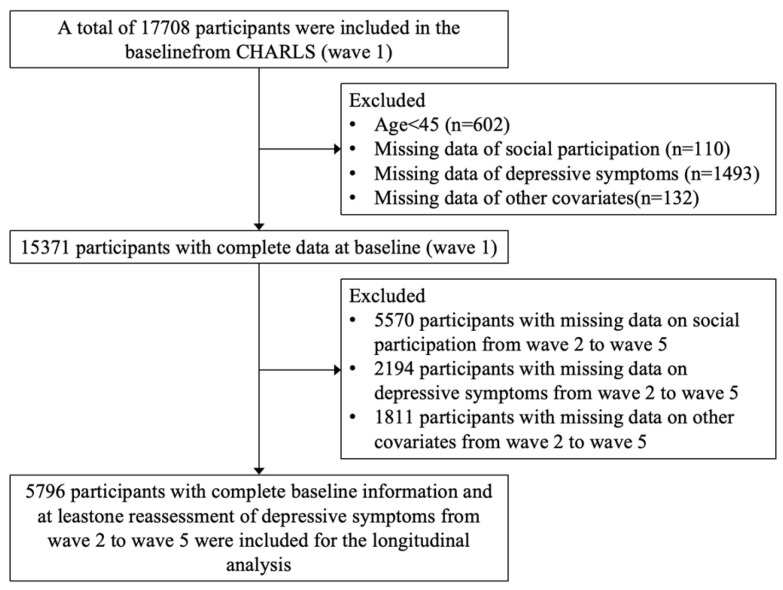
Flow diagram of sample selection from CHARLS waves 1–5 (2011–2020).

This study uses data from CHARLS. CHARLS is a large-scale and nationally representative survey project managed by the National Institute of Development of Peking University. It uses PPS multi-stage sampling and covers 150 counties and 450 communities in 28 provincial regions of China [27]. A total of 17,708 respondents were interviewed at baseline (2011), followed by follow-ups in 2013, 2015, 2018 and 2020. After excluding the participants whose baseline age is <45 years old and missing data, the final analysis sample was 5796 participants. (Figure 1).

### 2.2. Measures

#### 2.2.1. Depressive Symptoms

Depressive symptoms were measured in five survey waves using 10-item Center for Epidemiologic Studies Depression Scale (CESD-10), and its psychometric properties had been verified in the CHARLS cohort before (Cronbach’s α ≈ 0.78–0.79) [28]. Respondents indicated how frequently, over the previous week, they had experienced eight negatively worded symptoms: feeling bothered, depressed, lonely, or fearful; restless sleep; difficulty concentrating; a sense that everything was an effort; and inability to get going, together with two positively worded items capturing hopefulness about the future and happiness, both reverse-coded prior to summation. Each item uses four-point frequency anchor points with a score of 0–3 to obtain a comprehensive value of 0 to 30, where higher values indicate a greater symptom burden. Following thresholds adopted in earlier CHARLS-based work, a score of ≥12 was used to flag clinically meaningful depressive symptoms [29]. The specific items of CESD-10 are shown in Table S1.

#### 2.2.2. Social Participation

The level of social participation was operationalized from item DA056 of the CHARLS questionnaire, which records whether respondents had taken part in any of eight specified activities in the month preceding the interview. The activities covered in this item include socializing with friends; playing mahjong, chess, or cards, or spending time at community activity centers; offering help to relatives, friends, or neighbors; dancing, exercising, or engaging in similar pursuits in parks and other outdoor venues; taking part in events organized by the community; doing voluntary or charitable work, including unpaid care for the sick or disabled; enrolling in courses or training programs; and using the internet. Each activity was recorded dichotomously (1 = yes, 0 = no), and an overall participation score was generated by summing the number of activities endorsed. In line with prior work using these data, this count was then collapsed into an ordered three-level variable: no participation (0), engagement in a single activity (1), and engagement in two or more activities (≥2) [30].

#### 2.2.3. Covariates

Based on previous studies, the following covariates potentially associated with depression were included [31,32]. Sociodemographic characteristics included age (0 = 45–59 years, 1 = ≥60 years), sex (0 = male, 1 = female), marital status (0 = unmarried, 1 = married), place of residence (0 = rural, 1 = urban), and educational attainment (1 = illiterate, 2 = primary school or below, 3 = junior high school, 4 = senior high school or above). Health status was indexed by self-rated health (1 = good, 2 = fair, 3 = poor). Health-related behaviors comprised current smoking (0 = no, 1 = yes) and alcohol consumption (0 = no, 1 = yes). Functional disability was evaluated using the Katz Activities of Daily Living (ADL). The ADL in the CHARLS sample was excellent, with a Cronbach’s α of 0.703, and the value of KMO was 0.883 [31]. Participants were evaluated using the ADL scale and classified as none, mild, or severe, corresponding to disability in 0, 1–2, and ≥3 ADL items (1 = None, 2 = Mild, 3 = Severe).

### 2.3. Statistical Analysis

Several methods are available for analyzing longitudinal trajectories, including Growth Mixture Models (GMMs) and Latent Class Growth Analysis (LCGA). By contrast, Group-Based Trajectory Modeling (GBTM) is a data-driven, person-centered exploratory approach that offers greater model parsimony and convergence stability in large-scale nationally representative survey datasets [33,34]. GBTM employs maximum likelihood estimation to identify clusters of individuals exhibiting similar trajectories, thereby delineating several subgroups with distinct trajectory patterns from the overall population. Rather than fitting a single mean model to the study population, this approach estimates the probabilities of multiple trajectories, thereby preventing inter-individual heterogeneity from being obscured [35]. In view of the expected heterogeneity of the depressive syndrome trajectory of the study subjects during the 10-year observation period, GBTM was selected as the most suitable analysis method for this study.

In the present study, GBTM was used to identify the categories of middle-aged and elderly depressive symptoms. Stata commands traj and trajplot were used to analyze and visualize the resulting trajectory. The survey year served as the time scale, which is modeled using the normal distribution of the review of CESD-10 scores in 2011, 2013, 2015, 2018 and 2020. Model fitting commenced with a smaller number of subgroups, each initialized with higher-order polynomial terms; non-significant higher-order terms were sequentially removed, and lower-order functions were then fitted. The optimal model was selected on the basis of the Bayesian Information Criterion (BIC), the Akaike Information Criterion (AIC), and the Average Posterior Probability (AvePP). Lower BIC and AIC values indicate superior model fit, an AvePP > 0.7 denotes acceptable model adequacy, and each trajectory group was required to contain more than 5% of the observations [36].

The baseline characteristics of the depressive syndrome trajectory were compared using the variance analysis of continuous variables (ANOVA) and the chi-square test of classified variables. The polynomial logical regression model was used to check the relationship between the number of social activities and the trajectory of depression, and gender-stratified analyses (male vs. female) were conducted to assess the interaction effects on these trajectories. All data analysis is carried out using Stata MP 17, and the statistical significance is set to *p* < 0.05. All pictures are drawn using R (version 4.4.3).

## 3. Results

### 3.1. Trajectories of Depressive Symptoms

Based on GBTM, we determined the optimal number of trajectories characterizing the heterogeneity in depressive symptom scores observed across the 10-year follow-up. Table S2 presents the model fit statistics. Following the GBTM selection criteria, a four-trajectory solution emerged as the best fit, yielding four distinct patterns of depressive symptom progression. The AvePP for the four groups were 0.849, 0.797, 0.824, and 0.873, respectively, and each trajectory comprised more than 5% of the total analytical sample.

**Figure 2:**
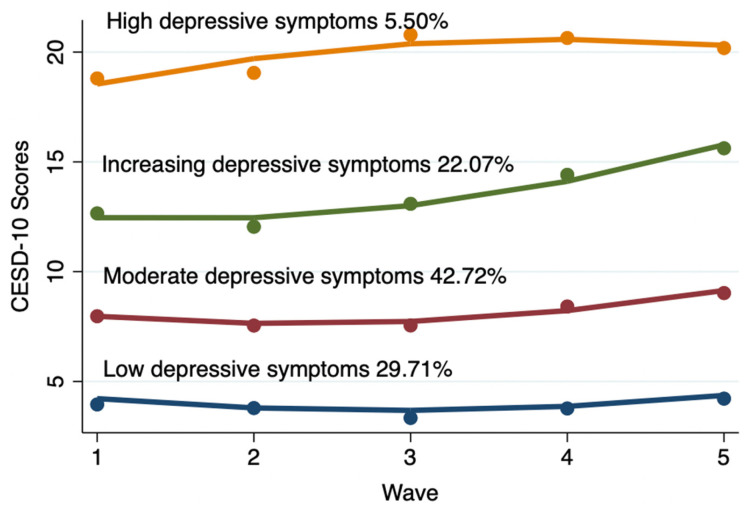
Trajectories of depressive symptoms from 2011 to 2020. Note: Percentages indicate the proportion of the total sample (*n* = 5796) assigned to each trajectory group based on GBTM across all five waves.

Based on the modeling results, four heterogeneous trajectory patterns emerged, which we labeled as follows: low depressive symptoms (*n* = 1722; 29.71%), moderate depressive symptoms (*n* = 2476; 42.72%), increasing depressive symptoms (*n* = 1279; 22.07%), and high depressive symptoms (*n* = 319; 5.50%), as shown in Figure 2.

### 3.2. Baseline Characteristics

Of the 5796 participants, 1921 (33.14%) were male and 3875 (66.86%) were female, with females constituting the majority of the sample. The mean CESD-10 score was 8.42 ± 6.26, with females (9.10 ± 6.48) scoring higher than males (7.07 ± 5.55), as shown in Table S3. The majority of participants were aged 45–59 years (66.70%), resided in urban areas (65.23%), had completed primary school education (39.84%), were married (86.18%), non-smokers (84.44%), non-drinkers (74.57%), reported their self-reported health as fair (50.90%), and had no ADL disability (85.23%). A total of 2885 participants (49.78%) reported engaging in at least one social activity: 1927 (33.25%) reported participation in one activity, and 958 (16.53%) reported participation in two or more activities. Significant differences across the trajectory groups were observed in age, sex, educational attainment, marital status, living residence, smoking status, drinking status, self-reported health, ADL and CESD-10 score. Detailed data are shown in Table 1.

### 3.3. Association Between Social Participation and Depressive Symptom Trajectories

**Figure 3:**
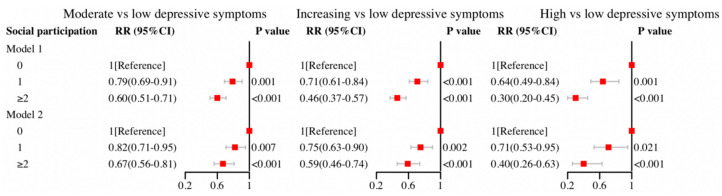
Association between social participation and depressive symptom trajectories. Model 1: crude model. Model 2: adjusted for age, sex, educational attainment, marital status, place of residence, smoking status, alcohol consumption, self-rated health, and functional disability.

Relative to middle-aged and older adults who engaged in no social activities, participation in a single activity was associated with a significantly reduced risk of being in the moderate (RR = 0.82; 95% CI: 0.71–0.95), increasing (RR = 0.75; 95% CI: 0.63–0.90), and high (RR = 0.71; 95% CI: 0.53–0.95) symptom trajectories (all *p* < 0.05). The protective effect was even more pronounced for participation in two or more activities, with corresponding risk ratios of 0.67 (95% CI: 0.56–0.81), 0.59 (95% CI: 0.46–0.74), and 0.40 (95% CI: 0.26–0.63) for the three adverse trajectories, respectively (all *p* < 0.05). Detailed data are shown in Figure 3.

### 3.4. Gender Differences Between Social Participation and Depressive Symptom Trajectories

**Figure 4:**
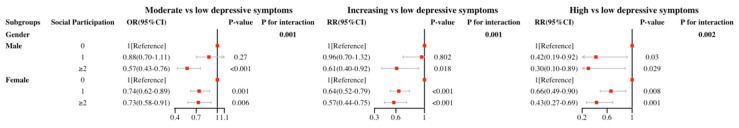
Gender-stratified analysis of the association between social participation and depressive symptom trajectories. Estimates were adjusted for age, educational attainment, marital status, place of residence, smoking status, alcohol consumption, self-rated health, and functional disability.

According to the analysis of gender stratification, there is a significant difference between the protective effect of social participation on the trajectory of adverse depression symptoms in males and females (*p* for interaction = 0.001, 0.001, and 0.002 for the moderate, increasing, and high trajectories, respectively). Among males, participation in a single activity reduced the risk only for the high trajectory (RR = 0.42; 95% CI: 0.19–0.92), with no significant effect on the moderate or increasing trajectories; a protective effect across all three trajectories emerged only with participation in two or more activities. Among females, even a single activity was associated with significantly reduced risk across all three trajectories, (all *p* < 0.01), and the protective effect strengthened with participation in two or more activities; this is presented in Figure 4.

### 3.5. Sensitivity Analysis

In the sensitivity analyses, we carried out several additional checks to confirm the robustness of our findings. Participants with memory-related diseases at the 2011 baseline were excluded, and the association between social participation and depressive symptom trajectories was re-estimated to minimize recall bias. The results were largely unchanged (Tables S4 and S5).

To further verify that the primary findings were not an artifact of the trajectory-based outcome specification, an additional robustness check was conducted using continuous CESD-10 scores as the outcome in an ordinary least squares regression model with the same covariates. Compared with no participation, engaging in one activity (β = −0.456, 95% CI: −0.772 to −0.141, *p* = 0.005) and two or more activities (β = −1.326, 95% CI: −1.735 to −0.918, *p* < 0.001) were both associated with significantly lower depressive symptom scores, confirming a dose–response gradient consistent with the primary analysis, as detailed in Table S6.

## 4. Discussion

### 4.1. Identification of Depressive Symptom Trajectories

This study identified four trajectories of depressive symptoms: low depressive symptoms, moderate depressive symptoms, increasing depressive symptoms, and high depressive symptoms, which are consistent with previous studies [37,38,39]. A total of 29.71% of middle-aged and older adults were classified as the low-depressive-symptoms group, with CESD-10 scores remaining consistently low throughout the follow-up period, far below the depressive symptom threshold (<12). A total of 42.72% of participants were placed into the moderate-depressive-symptoms group, where CESD-10 scores were relatively stable and remained below the clinical threshold, though showing a slight upward trend in later follow-up waves. The increasing depressive symptoms group comprised 22.07% of participants, with baseline CESD-10 scores already exceeding the threshold and showing a continuous and significant increase over subsequent follow-ups. The high-depressive-symptoms group accounted for 5.50% of participants, with CESD-10 scores remaining persistently elevated throughout the observation period, significantly above the clinical threshold and relatively stable. Compared with the study by You et al. [40], which focused on disabled middle-aged and older adults, the low- and high-depressive-symptoms groups identified in that study exhibited similar symptom patterns to those in the present study. However, the proportion of participants in the increasing-symptoms group was higher in our study than in theirs (22.07% vs. 18.41%), which may be attributable to our sample including general middle-aged and older adults without disabilities.

Earlier studies based on CHARLS data have similarly identified four trajectories: persistently low, increasing, decreasing, and persistently high [41]. The absence of a decreasing trajectory in our analysis may be attributable to the fact that the data came from the latest CHARLS dataset, which includes data from the fifth follow-up; this makes the trajectory analysis more robust and renders the study’s findings more applicable to the Chinese population.

### 4.2. Protective Effects of Social Participation on Depressive Symptom Trajectories

The core findings of this study are consistent with the existing longitudinal evidence. Social participation is a protective factor for middle-aged and older adult depressive symptoms [42]. In Europe, Croezen et al. analyzed the Survey of Health, Ageing and Retirement in Europe (SHARE) and found that specific forms of social participation were independently linked to reduced depressive symptoms over time, with consistent patterns across European regions [43]. Similarly, a cohort study based on the Japan Gerontological Evaluation Study (JAGES) showed that the relationship between social participation and depressive symptoms varies according to individual characteristics. Promoting social participation, especially for the elderly with low socioeconomic status, can improve mental health and reduce mental health disparities [44]. From a theoretical standpoint, this finding resonates with the central tenet of social identity theory, which holds that the sense of social identity derived from belonging to a particular social group serves as a vital intrinsic resource for sustaining mental health [45]. The loss of social roles after retirement will damage the mental health of middle-aged and older adults, whereas social participation enables them to reconstruct their social identities and gain access to emotional support networks, thereby effectively buffering the psychological strain stemming from role loss or shifts in family structure [46]. Collectively, our findings align with this international evidence. This suggests that the protective role of social participation in late-life depression may come from universal psychosocial mechanisms that go beyond cultural boundaries, while the magnitude and patterning of these effects may be shaped by local social structures and norms.

Of particular note, the protective effect of participating in two or more activities was significantly stronger than that of participating in only one activity, suggesting that the benefits of different forms of participation may be additive or synergistic [47,48,49]. This pattern is corroborated by evidence from other national contexts. In the United States, Jeon and Charles demonstrated using longitudinal data from the Health and Retirement Study (HRS) that greater variety of social activities was a significant protective factor against depressive symptoms at follow-up [50]. In Korea, Roh et al. reported a dose–response relationship in a three-year community-based cohort, whereby participation in two or three types of activities was associated with substantially lower odds of depression compared to participation in only one type [51]. Consistent with role accumulation theory, engaging in diverse types of activities helps maintain multiple social roles [52]. Among middle-aged and older adults, the sense of altruistic value gained through volunteering [53], along with the improvements in health and cognitive function derived from physical exercise, contribute to a reduced risk of depression [54].

### 4.3. Gender Differences in the Protective Effects of Social Participation on Depressive Symptom Trajectories

Gender-stratified analyses uncovered substantial heterogeneity in the effects of social participation across population subgroups, with these differences confirmed as statistically significant by interaction tests (all interaction effects *p* < 0.05). Females made up a larger share of the increasing- and high-symptom trajectories. This indicates that older females have a higher risk of depression, and this finding agrees with earlier studies [55]. From a biopsychological perspective, the fluctuations in sex hormones that females experience across the life course heighten their biological susceptibility to psychosocial stressors, thereby elevating the risk of depression [56]. From a sociological perspective, given that males and females occupy distinct social roles, females continue to assume caregiving responsibilities into later life, rendering them more vulnerable than males to the heavy burdens of caregiving duties and the multifaceted stresses they entail [23]. These vulnerabilities are further compounded by lifestyle transitions characteristic of middle and later adulthood. Females are more likely to retire early to take on family caregiving roles, with significant gender differences in retirement pathways [57]. After retirement, they often face a further intensification of domestic roles, including grandchild care [58] and spousal caregiving [59], which sustains chronic stress exposure and elevates the risk of depression. Furthermore, due to gender differences in life expectancy, older women experience widowhood much more often than older men do. Widowhood has a serious negative impact on the mental health of elderly women and is directly related to the risk of depression [60].

With respect to the protective mechanisms of social participation, our study found that participation in just a single-activity-conferred significant protection against all adverse trajectories among females, whereas males required engagement in ≥2 activities to attain comprehensive protection. This difference may be attributed to the different lifestyle transitions experienced by middle-aged and older adults of different genders, which shape the mechanisms through which they benefit from social participation. For middle-aged and older men, their social networks are largely constructed around work-related relationships, and life after retirement is often accompanied by the abrupt loss of occupational identity, structured daily routines, and achievement-based social hierarchies; men may perceive retirement as a social failure [61]. For example, a study from Australia showed that the effect of retirement on psychological distress was only seen in men, while women were not affected by retirement in that regard [62]. Consistent with this lifestyle context, men rely more on instrumental social support, which typically requires diverse activity contexts and higher participation frequency to be fully realized; therefore, they benefit more from the diversity and frequency of participation [63]. In contrast, across the life course, older women consistently maintain broader and more emotionally expressive social networks than men [64]. Evidence from gender-stratified analyses of middle-aged and older populations consistently shows that emotional support is associated with mental health only in women [65], whereas men’s health is more dependent on the breadth and structure of social networks [66]. Women tend to prefer emotional support, which can be obtained through a single, intimate interaction [67]. Therefore, females derive benefits more efficiently than males from single-activity participation [68], achieving health protection at a lower threshold of engagement [69].

### 4.4. Limitations

This study has several limitations. The CESD-10 scale used in CHARLS to assess depressive symptoms among middle-aged and older adults relies on self-report and may therefore be susceptible to recall bias. In addition, although this study drew on a large sample from a reliable database, and attrition analysis showed no significant difference in baseline depressive symptoms between included and excluded participants, the exclusion of participants who did not meet the eligibility criteria may still introduce a degree of selection bias due to practical constraints during follow-up, and participant attrition may also lead to healthy survivor bias. Lastly, many factors influence depression in middle-aged and older adults, and our study may not have included all of the relevant determinants.

## 5. Conclusions

This study indicates that increased social participation significantly alleviates depressive symptoms in middle-aged and older adults, with the protective effect showing marked gender differences (all interaction effects *p* < 0.05). Specifically, among women, even participation in a single social activity provided broad and stable protection across moderate-, increasing-, and high-symptom trajectories. Among men, higher levels of participation (≥2 activities) were required to provide significant protection across all adverse trajectories, while participation in a single activity was effective only for the high-depression-symptom trajectory.

These findings underscore the importance of fully considering gender-specific moderating effects when designing interventions for depressive symptoms. For middle-aged and older women, priority should be given to improving the accessibility and convenience of social participation. This can be achieved by establishing community-based support groups to facilitate mutual emotional exchange, organizing low-threshold collective activities such as interest groups, mutual aid networks, and volunteer services, or developing intergenerational programs that pair older women with grandchildren, while providing flexible scheduling to accommodate caregiving responsibilities. Emphasis should be placed on conducting regular depressive symptom assessments for women who are empty-nest, widowed, or undertaking family caregiving responsibilities and establishing dedicated psychological support hotlines to provide emotional assistance. These initiatives can help alleviate chronic stress associated with caregiving roles and widowhood, while simultaneously rebuilding a sense of social identity. For middle-aged and older men, further efforts are needed to promote greater diversity and depth of participation. Targeted strategies may include developing structured volunteer programs that leverage occupational expertise, such as skills training and technical consulting, and encouraging simultaneous participation in community governance roles to help reconstruct the occupational identity lost at retirement. By integrating these gender-sensitive, population-tailored approaches into community mental health programs, policymakers and practitioners can more effectively reduce the burden of late-life depression and narrow gender-related mental health disparities.

## Figures and Tables

**Table 1:** Baseline characteristics of the total sample and the four depressive symptom trajectory subsamples.

Characteristics	Total Sample (*n* = 5796) *n* (%)	Low Depressive Symptoms (*n* = 1722) *n* (%)	Moderate Depressive Symptoms (*n* = 2476) *n* (%)	Increasing Depressive Symptoms (*n* = 1279) *n* (%)	High Depressive Symptoms (*n* = 319) *n* (%)	*p* Value
Sex						<0.001
Male	1921 (33.14)	765 (44.43)	824 (33.28)	287 (22.44)	45 (14.11)	
Female	3875 (66.86)	957 (55.57)	1652 (66.72)	992 (77.56)	274 (85.89)	
Age group						0.001
45–59	3866 (66.70)	1201 (69.74)	1653 (66.76)	819 (64.03)	193 (60.50)	
≥60	1930 (33.30)	521 (30.26)	823 (33.24)	460 (35.97)	126 (39.50)	
Living residence						<0.001
Urban	2015 (34.77)	799 (46.40)	830 (33.52)	321 (25.10)	65 (20.38)	
Rural	3781 (65.23)	923 (53.60)	1646 (66.48)	958 (74.90)	254 (79.62)	
Educational level						<0.001
Illiterate	1586 (27.36)	323 (18.76)	641 (25.89)	480 (37.53)	142 (44.51)	
Primary school	2309 (39.84)	613 (35.60)	1043 (42.12)	522 (40.81)	131 (41.07)	
Middle or high school	1271 (21.93)	485 (28.16)	545 (22.01)	204 (15.95)	37 (11.60)	
College or above	630 (10.87)	301 (17.48)	247 (9.98)	73 (5.71)	9 (2.82)	
Marital status						<0.001
Married	4995 (86.18)	1544 (89.66)	2149 (86.79)	1056 (82.56)	246 (77.12)	
Unmarried	801 (13.82)	178 (10.34)	327 (13.21)	223 (17.44)	73 (22.88)	
Self-reported health						<0.001
Good	1349 (23.27)	683 (39.66)	516 (20.84)	139 (10.87)	11 (3.45)	
Fair	2950 (50.90)	874 (50.76)	1357 (54.81)	623 (48.71)	96 (30.09)	
Poor	1497 (25.83)	165 (9.58)	603 (24.35)	517 (40.42)	212 (66.46)	
Smoking status						<0.001
No	4894 (84.44)	1429 (82.98)	2063 (83.32)	1112 (86.94)	290 (90.91)	
Yes	902 (15.56)	293 (17.02)	413 (16.68)	167 (13.06)	29 (9.09)	
Drinking status						<0.001
No	4322 (74.57)	1191 (69.16)	1828 (73.83)	1039 (81.24)	264 (82.76)	
Yes	1474 (25.43)	531 (30.84)	648 (26.17)	240 (18.76)	55 (17.24)	
ADL status						<0.001
None	4940 (85.23)	1617 (93.90)	2155 (87.04)	985 (77.01)	183 (57.37)	
Mild	610 (10.52)	69 (4.01)	240 (9.69)	209 (16.34)	92 (28.84)	
Severe	246 (4.24)	36 (2.09)	81 (3.27)	85 (6.65)	44 (13.79)	
Social participation						<0.001
0	2911 (50.22)	746 (43.32)	1274 (51.45)	702 (54.89)	189 (59.25)	
1	1927 (33.25)	594 (34.49)	816 (32.96)	415 (32.45)	102 (31.97)	
≥2	958 (16.53)	382 (22.18)	386 (15.59)	162 (12.67)	28 (8.78)	
CESD-10 score, mean ± SD	8.42 ± 6.26	3.63 ± 3.17	8.04 ± 4.73	12.91 ± 5.70	19.26 ± 5.48	<0.001

Note: Sample size *n* = 5796.

## Data Availability

The original data presented in this study are openly available at http://charls.pku.edu.cn/en (accessed on 15 April 2026). The datasets used and analyzed during the current study are available from the corresponding author on reasonable request.

## Supplementary Materials

The following supporting information can be downloaded at https://www.mdpi.com/article/10.3390/healthcare14131845/s1, Table S1. Items for calculating CESD-10; Table S2. Fit statistics of group-based trajectory analysis; Table S3. Baseline characteristics of middle-aged and older adults by gender (*n* = 5796); Table S4. Association between social participation and depressive symptom trajectories after excluding participants who had memory-related diseases at the 2011 baseline (*n* = 5734); Table S5. Gender-stratified analysis of the association between social participation and depressive symptom trajectories after excluding participants who had memory-related diseases at the 2011 baseline (*n* = 5734); Table S6. Association between social participation and continuous depressive symptom scores (OLS regression) (*n* = 5796); Table S7. Analysis of attrition between included and excluded participants.

## Abbreviations

The following abbreviations are used in this manuscript:

CHARLS	China Health and Retirement Longitudinal Study
GBTM	Group-Based Trajectory Modeling
