# Supplementary material for: Gender Differences in the Protective Effects of Social Participation on Depressive Symptom Trajectories Among Middle-Aged and Older Adults in China: A Nationwide Longitudinal Study
Source: Healthcare (Basel). 2026 Jun 25;14(13):1845. doi: 10.3390/healthcare14131845 (PMC13361681; doi:10.3390/healthcare14131845)
Supplement: Supplementary file 1 [file healthcare-14-01845-s001.zip › healthcare-4320118-supplementary.pdf]

Supplementary Material

Table S1. Items for calculating CESD-10

Items	Definition	Score
1. Bothered by little things	Rarely or none of the time (< 1 day)	0
	Some or a little of the time (1-2 days)	1
	Occasionally or a moderate amount of the time (3 days)	2
	Most or all of the time (5-7 days)	3
2. Had trouble keeping my mind on what I was doing	Rarely or none of the time (< 2 day)	0
	Some or a little of the time (1-3 days)	1
	Occasionally or a moderate amount of the time (4 days)	2
	Most or all of the time (5-8 days)	3
3. Felt depressed	Rarely or none of the time (< 3 day)	0
	Some or a little of the time (1-4 days)	1
	Occasionally or a moderate amount of the time (5 days)	2
	Most or all of the time (5-9 days)	3
4. Felt that everything I did was an effort	Rarely or none of the time (< 4 day)	0
	Some or a little of the time (1-5 days)	1
	Occasionally or a moderate amount of the time (6 days)	2
	Most or all of the time (5-10 days)	3
5. Felt hopeful about the future	Rarely or none of the time (< 5 day)	3
	Some or a little of the time (1-6 days)	2
	Occasionally or a moderate amount of the time (7 days)	1
	Most or all of the time (5-11 days)	0
6. Felt fearful	Rarely or none of the time (< 6 day)	0
	Some or a little of the time (1-7 days)	1
	Occasionally or a moderate amount of the time (8 days)	2
	Most or all of the time (5-12 days)	3
7. Sleep was restless	Rarely or none of the time (< 7 day)	0
	Some or a little of the time (1-8 days)	1
	Occasionally or a moderate amount of the time (9 days)	2
	Most or all of the time (5-13 days)	3
8. Was happy	Rarely or none of the time (< 8 day)	3
	Some or a little of the time (1-9 days)	2
	Occasionally or a moderate amount of the time (10 days)	1
	Most or all of the time (5-14 days)	0
9. Felt lonely	Rarely or none of the time (< 9 day)	0
	Some or a little of the time (1-10 days)	1
	Occasionally or a moderate amount of the time (11 days)	2
	Most or all of the time (5-15 days)	3
10. Could Not Get Going	Rarely or none of the time (< 10 day)	0
	Some or a little of the time (1-11 days)	1
	Occasionally or a moderate amount of the time (12 days)	2
	Most or all of the time (5-16 days)	3

Notes: CESD-10, the ten-item Center for Epidemiological Studies Depression Scale.

Table S2. Fit statistics of group-based trajectory analysis

Fit statistics	Number of class				
	1	2	3	4	5
BIC	-92177.35	-88366.56	-87480.63	-87284.05	-87239.37
AIC	-92164.02	-88339.9	-87443.98	-87230.73	-87156.05
Entropy	—	0.818	0.767	0.704	0.680
Class proportion, %					
Class 1	100	67.67	47.33	29.71	21.00
Class 2		32.33	40.68	42.72	41.49
Class 3			11.99	22.07	25.26
Class 4				5.50	10.04
Class 5					2.21
Average posterior probabilities					
Class 1	1	0.958	0.911	0.849	0.796
Class 2		0.923	0.864	0.797	0.764
Class 3			0.900	0.824	0.768
Class 4				0.873	0.795
Class 5					0.838

Table S3. Baseline characteristics of middle-aged and older adults by gender(*n*=5796)

Characteristics	Total sample *n*(%)	Male *n*(%)	Female *n*(%)	P value
Age group				
45-59	3866 (66.70)	1207 (31.22)	2659 (68.78)	<0.001
≥60	1930 (33.30)	714 (36.99)	1216 (63.01)	
Living residence				
Urban	2015 (34.77)	633 (31.41)	1382 (68.59)	0.041
Rural	3781 (65.23)	1288 (34.06)	2493 (65.94)	
Educational level				
Illiterate	1586 (27.36)	186 (11.73)	1400 (88.27)	<0.001
Primary school	2309 (39.84)	847 (36.68)	1462 (63.32)	
Middle or high school	1271 (21.93)	573 (45.08)	698 (54.92)	
College or above	630 (10.87)	315 (50.00)	315 (50.00)	
Marital status				
Married/cohabiting	4995 (86.18)	1738 (34.79)	3257 (65.21)	<0.001
Unmarried/separated	801 (13.82)	183 (22.85)	618 (77.15)	
Self-reported health				
Good	1349 (23.27)	535 (39.66)	814 (60.34)	<0.001
Fair	2950 (50.90)	978 (33.15)	1972 (66.85)	
Poor	1497 (25.83)	408 (27.25)	1089 (72.75)	
Smoking status				
No	4894 (84.44)	1129 (23.07)	3765 (76.93)	<0.001
Yes	902 (15.56)	792 (87.80)	110 (12.20)	
Drinking status				
No	4322 (74.57)	901 (20.85)	3421 (79.15)	<0.001
Yes	1474 (25.43)	1020 (69.20)	454 (30.80)	
ADL status				
No difficulty	4940 (85.23)	1691 (34.23)	3249 (65.77)	<0.001
Some difficulty	610 (10.52)	165 (27.05)	445 (72.95)	
Severe difficulty	246 (4.24)	65 (26.42)	181 (73.58)	
Social participation				
0	2911 (50.22)	978 (33.60)	1933 (66.40)	0.008
1	1927 (33.25)	594 (30.83)	1333 (69.17)	
≥2	958 (16.53)	349 (36.43)	609 (63.57)	
CES-D score mean ± SD	8.42 ± 6.26	7.07 ± 5.55	9.10 ± 6.48	<0.001

Table S4. Association between social participation and depressive symptoms trajectories after excluding participants who had memory-related diseases at the 2011 baseline(*n* = 5734)

Moderate vs low depressive symptoms				
Social participation	Model 1		Model 2	
	RR (95% CI)	p-value	RR (95% CI)	p-value
1	0.82 (0.71, 0.95)	0.002	0.82 (0.71, 0.95)	0.002
≥2	0.68 (0.57, 0.82)	<0.001	0.68 (0.57, 0.82)	<0.001
**Increasing vs low depressive symptoms**				
Social participation	Model 1		Model 2	
	RR (95% CI)	p-value	RR (95% CI)	p-value
1	0.76 (0.64, 0.91)	0.003	0.76 (0.64, 0.91)	0.003
≥2	0.59 (0.46, 0.74)	<0.001	0.59 (0.46, 0.74)	<0.001
**High vs low depressive symptoms**				
Social participation	Model 1		Model 2	
	RR (95% CI)	p-value	RR (95% CI)	p-value
1	0.69 (0.51, 0.92)	0.013	0.69 (0.51, 0.92)	0.013
≥2	0.41 (0.27, 0.65)	<0.001	0.41 (0.27, 0.65)	<0.001

Table S5. Gender-stratified analysis of the association between social participation and depressive symptoms trajectories after excluding participants who had memory-related diseases at the 2011 baseline(n = 5734)

Variables		Moderate vs low depressive symptoms		Increasing vs low depressive symptoms		High vs low depressive symptoms	
		RR (95%CI)	P of the interaction	RR (95%CI)	P of the interaction	RR (95%CI)	P of the interaction
Gender	Social participation						
Male	1	0.88 (0.70, 1.11)	0.000	0.92 (0.66, 1.27)	0.000	0.42 (0.19, 0.93)	0.000
	≥2	0.58 (0.44, 0.77)		0.60 (0.40, 0.91)		0.31 (0.10, 0.94)	
Female	1	0.75 (0.62, 0.90)		0.66 (0.54, 0.81)		0.64 (0.47, 0.87)	
	≥2	0.73 (0.58, 0.91)		0.57 (0.44, 0.76)		0.44 (0.27, 0.71)	

Table S6. Association between social participation and continuous depressive symptoms scores (OLS regression)(n = 5796)

Social participation	Model 1 (Unadjusted)			Model 2 (Adjusted)		
	β	95% CI	p-value	β	95% CI	p-value
0 activities (ref)	—	—	—	—	—	—
1 activity	-0.615	(-0.973, -0.258)	0.001	-0.456	(-0.772, -0.141)	0.005
≥2 activities	-2.161	(-2.614, -1.707)	<0.001	-1.326	(-1.735, -0.918)	<0.001

Note: Model 1 is unadjusted. Model 2 is adjusted for age, sex, place of residence, educational attainment, marital status, self-rated health, smoking status, alcohol consumption, and functional disability. β denotes unstandardized regression coefficients.

Table S7. Analysis of attrition between included and excluded participants

Characteristics	Combined (*n* = 17708)	Excluded (*n* = 11,912)	Included (*n* = 5,796	P value
Age group				<0.001
45-59 years	10,243	6,377	3,866	
60+ years	7,465	5,535	1,930	
Sex				<0.001
Male	8,478	6,557	1,921	
Female	9,228	5,353	3,875	
Living residence				<0.001
Urban	7,171	5,156	2,015	
Rural	10,537	6,756	3,781	
Educational level				<0.001
Illiterate	4,851	3,265	1,586	
Primary school	6,921	4,612	2,309	
Middle or high school	3,677	2,406	1,271	
College or above	2,244	1,614	630	
Marital status				<0.001
Married/cohabiting	14,170	9,175	4,995	
Unmarried/separated	3,538	2,737	801	
Self-reported health				<0.001
Good	4,129	2,780	1,349	
Fair	8,551	5,601	2,950	
Poor	4,881	3,384	1,497	
Smoking status				<0.001
Non-current smokers	12,036	7,142	4,894	
Current smokers	4,871	3,969	902	
Drinking status				<0.001
Non-regular drinkers	11,785	7,463	4,322	
Regular drinkers	5,767	4,293	1,474	
ADL status				<0.001
No difficulty	14,478	9,538	4,940	
Some difficulty	1,976	1,366	610	
Severe difficulty	1,254	1,008	246	
Social participation				<0.001
0 (no participation)	9,523	6,612	2,911	
1 activity	5,354	3,427	1,927	
≥2 activities	2,719	1,761	958	
CES-D score, mean ± SD	8.40 ± 6.33	8.39 ± 6.37	8.42 ± 6.26	0.753

Note: The total number in the "Combined" column may vary slightly due to missing values in individual variables.
